# Hepatitis C Virus Modulates Solute carrier family 3 member 2 for Viral Propagation

**DOI:** 10.1038/s41598-018-33861-6

**Published:** 2018-10-19

**Authors:** Ngan N. T. Nguyen, Yun-Sook Lim, Lap P. Nguyen, Si C. Tran, Trang T. D. Luong, Tram T. T. Nguyen, Hang T. Pham, Han N. Mai, Jae-Woong Choi, Sang-Seop Han, Soon B. Hwang

**Affiliations:** 1 National Research Laboratory of Hepatitis C Virus, Hallym University, Anyang, South Korea; 2 Laboratory of RNA Viral Diseases, Korea Zoonosis Research Institute, Chonbuk National University, Iksan, South Korea

## Abstract

Hepatitis C virus (HCV) exploits an extensive network of host proteins to maintain chronic infection. Using RNA-Seq technology, we identified 30 host genes that were differentially expressed in cell culture grown HCV (HCVcc)-infected cells. Of these candidate genes, we selected solute carrier family 3 member 2 (SLC3A2) for further investigation. SLC3A2, also known as CD98hc, is a member of the solute carrier family and encodes a subunit of heterodimeric amino acid transporter. SLC3A2 and LAT1 constitute a heterodimeric transmembrane protein complex that catalyzes amino acid transport. In this study, we showed that HCV upregulated both mRNA and protein expression levels of SLC3A2 and this upregulation occurred through NS3/4A-mediated oxidative stress. HCV also elevated SLC3A2/LAT1 complex level and thus mammalian target of rapamycin complex 1 (mTORC1) signaling was activated. We further showed that L-leucine transport level was significantly increased in Jc1-infected cells as compared with mock-infected cells. Using RNA interference technology, we demonstrated that SLC3A2 was specifically required for the entry step but not for other stages of the HCV life cycle. These data suggest that SLC3A2 plays an important role in regulating HCV entry. Collectively, HCV exploits SLC3A2 for viral propagation and upregulation of SLC3A2 may contribute to HCV-mediated pathogenesis.

## Introduction

Hepatitis C virus (HCV) is one of the major prevalent pathogen of chronic liver diseases, including liver cirrhosis and hepatocellular carcinoma. Recently, it is estimated that approximately 80 million people worldwide are chronically infected with HCV^1^. HCV is an envelope virus with a positive-sense, single-stranded RNA that belongs to the genus *Hepacivirus* within the family *Flaviviridae*. The genome of HCV contains 9,600 nucleotides which encodes a single polyprotein of 3,010 amino acids, which is processed by cellular and viral proteases into three structural proteins (core, E1, and E2) and seven nonstructural (NS) proteins (p7, NS2, NS3, NS4A, NS4B, NS5A, and NS5B)^2^. Although numerous direct-acting antivirals (DAAs) are currently available to treat HCV patients, sustained virologic responses are still low in certain HCV genotypes. Moreover, the prices of DAAs are still too high and unaffordable for more than 95% of HCV patients worldwide^3^. Because of intrinsic nature of RNA genome of HCV, resistant-associated mutants to DAAs occur naturally due to low genetic barriers to drug resistance. Since viruses depend highly on host cellular proteins in all steps of the viral life cycle for their own propagation, host-targeting antivirals may have advantages in high genetic barriers to resistance and a potential for pan-genotypic antiviral activity. Therefore, blocking any step of the virus life cycle results in an efficient blockade of viral production and thus could be a potential target for viral therapy.

Solute carrier family 3 member 2 (SLC3A2), a gene encoding for a subunit of heterodimeric amino acid transporter, belongs to CD98 family and regulates intracellular amino acid levels. The CD98 family is composed of widely expressed cell surface disulfide-linked 125-kDa heterodimeric type II membrane glycoproteins containing an 80-kDa glycosylated heavy chain (SLC3A2) and a 45-kDa non-glycosylated light chain (LAT1)^4,5^. SLC3A2 consists of three domains, including the extracellular domain, the transmembrane domain, and the cytoplasmic domain^4^. SLC3A2 together with another subunit functions as an amino acid transporter and integrin signaling enhancer^6^. SLC3A2 is a glycosylated protein consisting of 529 amino acids^7,8^. SLC3A2 is required to protect the cells from oxidative stress through leucine transport via amino acid transporters^6^. Growing evidences show that SLC3A2 deletion induces strong impairment of cell proliferation both *in vitro* and *in vivo*, and it is overexpressed in highly proliferative cells in both physiological and pathological conditions^6–10^. Furthermore, changes in SLC3A2 levels potentially affect cell growth and survival through modulation of leucine-mediated mTOR activation^11^.

In an attempt to develop host-targeted agents (HTAs), we recently performed transcriptome sequencing (RNA-Seq) analysis and identified 30 host genes which were upregulated in HCV-infected cells^12^. In the present study, we selected SLC3A2 and showed that HCV upregulated SLC3A2 expression via oxidative stress. Furthermore, SLC3A2/LAT1 complex level was increased in the context of HCV replication, suggesting that HCV might regulate leucine transporter to promote cell proliferation via mammalian target of rapamycin complex 1 (mTORC1) signaling. We further showed that SLC3A2 was involved in the entry step of the HCV life cycle. These data suggest that HCV modulates SLC3A2 to provide a favorable milieu for viral propagation.

## Results

### HCV upregulates SLC3A2 expression level

**Figure 1:**
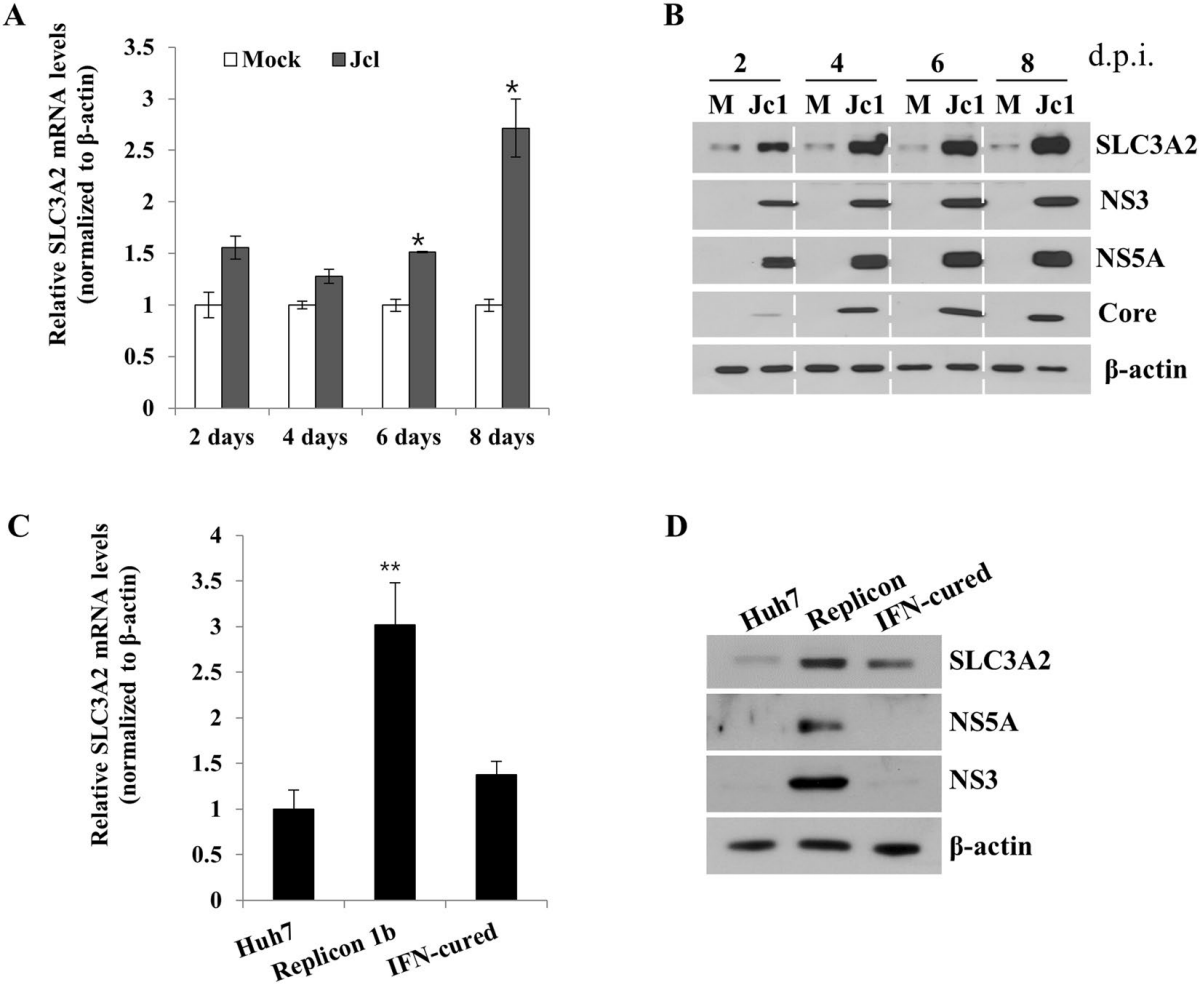
HCV upregulates SLC3A2 expression level. (**A**) Huh7.5 cells were either mock-infected or infected with HCV Jc1 for 4 h and then mRNA levels of SLC3A2 were analyzed by qRT-PCR at the indicated time points. (**B**) Total cell lysates harvested at the indicated time points were immunoblotted with the corresponding antibodies. M, mock. (**C**) mRNA levels of SLC3A2 in Huh7 cells, replicon cells derived from HCV genotype 1b, and IFN-cured cells were determined by qRT-PCR. (**D**) Protein levels of SLC3A2 in Huh7 cells, replicon cells derived from HCV genotype 1b, and IFN-cured cells were immunoblotted with the indicated antibodies. Data represent averages from at least three independent experiments for panels A and C. The asterisks indicate significant differences (*p < 0.05; **p < 0.01) from the value for the control.

Using RNA-Seq technology, we recently identified 30 host genes that were highly differentially expressed in the context of HCV infection^12^. Among these genes, we selected SLC3A2 to investigate the possible involvement of amino acid transporter function of SLC3A2 in HCV propagation. To validate the increase of SLC3A2 expression in HCVcc-infected cells, Huh7.5 cells infected with either mock or Jc1^13^ were harvested at 2-day intervals. We showed that mRNA levels of SLC3A2 were noticeably increased at day 6, and its levels were doubled at day 8 in Jc1-infected cells compared with the level in mock-infected cells (Fig. 1A). Consistently, protein level of SLC3A2 was also gradually increased during the course of HCV infection (Fig. 1B). Moreover, both mRNA (Fig. 1C) and protein (Fig. 1D) levels of SLC3A2 were considerably upregulated in Huh7 cells harboring HCV subgenomic replicon as compared to those in interferon-cured and parental Huh7 cells. These data clearly indicate that HCV upregulates SLC3A2 expression.

### HCV upregulates SLC3A2 expression via redox signaling

**Figure 2:**
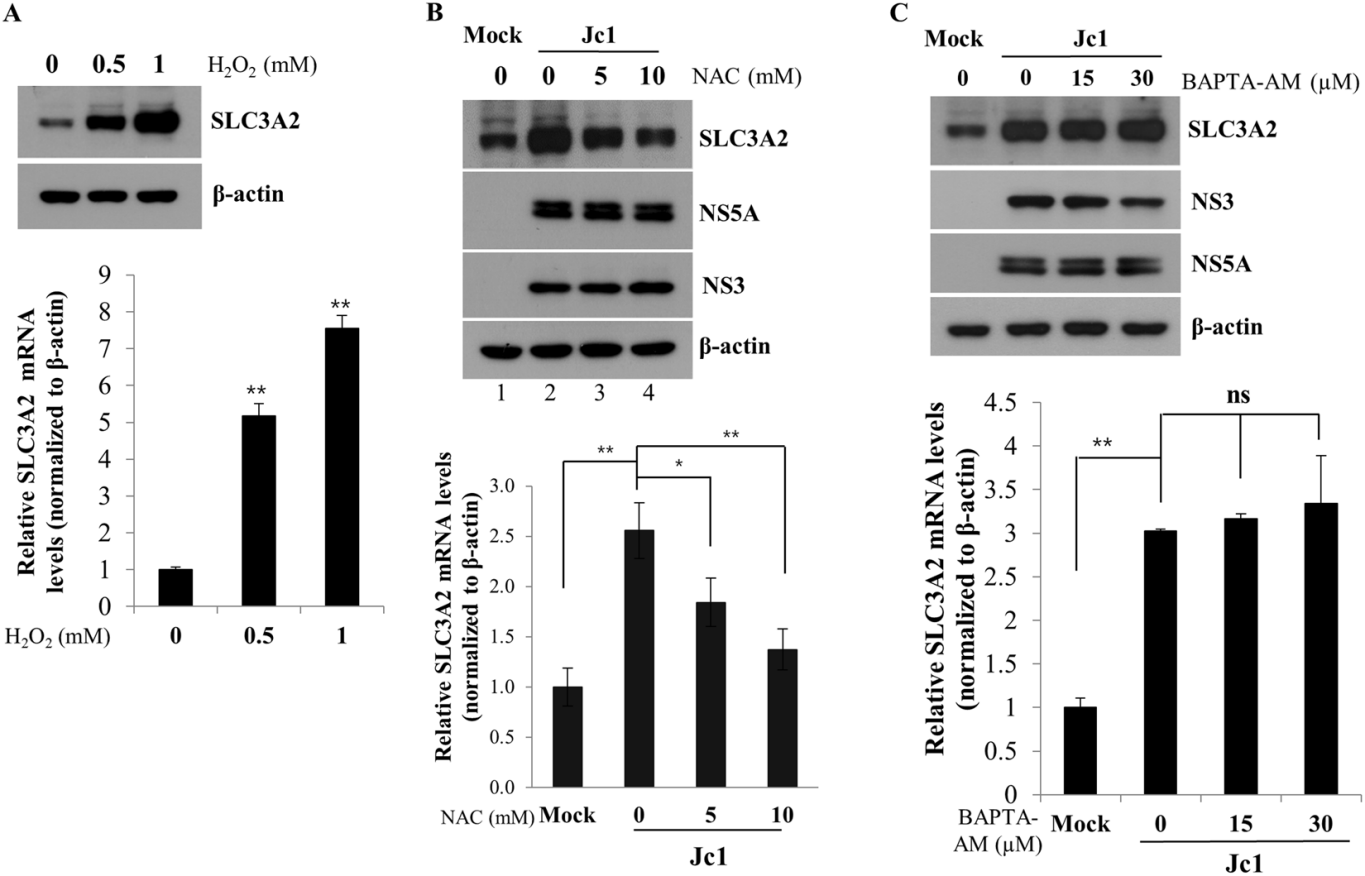
SLC3A2 expression is upregulated by HCV-induced oxidative stress. (**A**) Huh7.5 cells were either left untreated or treated with increasing amounts of H_2_O_2_. At 24 h after treatment, total cell lysates were immunoblotted with the indicated antibodies (upper panel) and mRNA levels of SLC3A2 were analyzed by qRT-PCR (bottom panel). (**B**) Huh7.5 cells were either mock infected or infected with HCV Jc1. At 6 days postinfection, cells were either left untreated or treated with increasing amounts of NAC. At 24 h after treatment, total cell lysates were immunoblotted with the indicated antibodies (upper panel) and mRNA levels of SLC3A2 were analyzed by qRT-PCR (bottom panel). (**C**) Huh7.5 cells were either mock-infected or infected with HCV Jc1. At 6 days postinfection, cells were either left untreated or treated with increasing amounts of BAPTA-AM. At 24 h after treatment, total cell lysates were immunoblotted with the indicated antibodies (upper panel) and mRNA levels of SLC3A2 were analyzed by qRT-PCR (bottom panel).

It has been previously reported that HCV regulates ROS signaling cascade, leading to oxidative stress^14–18^. It is also known that SLC3A2 protects cells from oxidative stress by activating amino acid transporter system^6^. To investigate if the reactive oxygen species (ROS) is involved in upregulation of SLC3A2 in HCV infected cells, Huh7.5 cells were treated with increasing amounts of H_2_O_2_ for 24 h and then both protein and mRNA levels of SLC3A2 were analyzed by either immunoblot analysis and qRT-PCR, respectively. Figure 2A shows that protein levels of SLC3A2 in Huh7.5 cells were gradually increased by H_2_O_2_. Consistently, mRNA levels of SLC3A2 were significantly increased by H_2_O_2_. To corroborate these results, Huh7.5 cells were either mock infected or infected with Jc1. The cells were then treated with either NAC, an antioxidant agent, or BAPTA-AM, an intracellular Ca^2+^ chelator. As expected, both protein and mRNA expression levels of SLC3A2 were increased by HCV infection (Fig. 2B, lane 2). However, HCV-induced SLC3A2 expression levels were gradually decreased by NAC in a dose-dependent manner (Fig. 2B, lanes 3 and 4). Meanwhile, BAPTA-AM displayed no effect on protein and mRNA expression levels of SLC3A2 in HCV-infected cells (Fig. 2C). Taken together, these data suggest that oxidative stress is involved in upregulation of SLC3A2 in the context of HCV infection.

### SLC3A2 expression level is upregulated by NS3/4A

**Figure 3:**
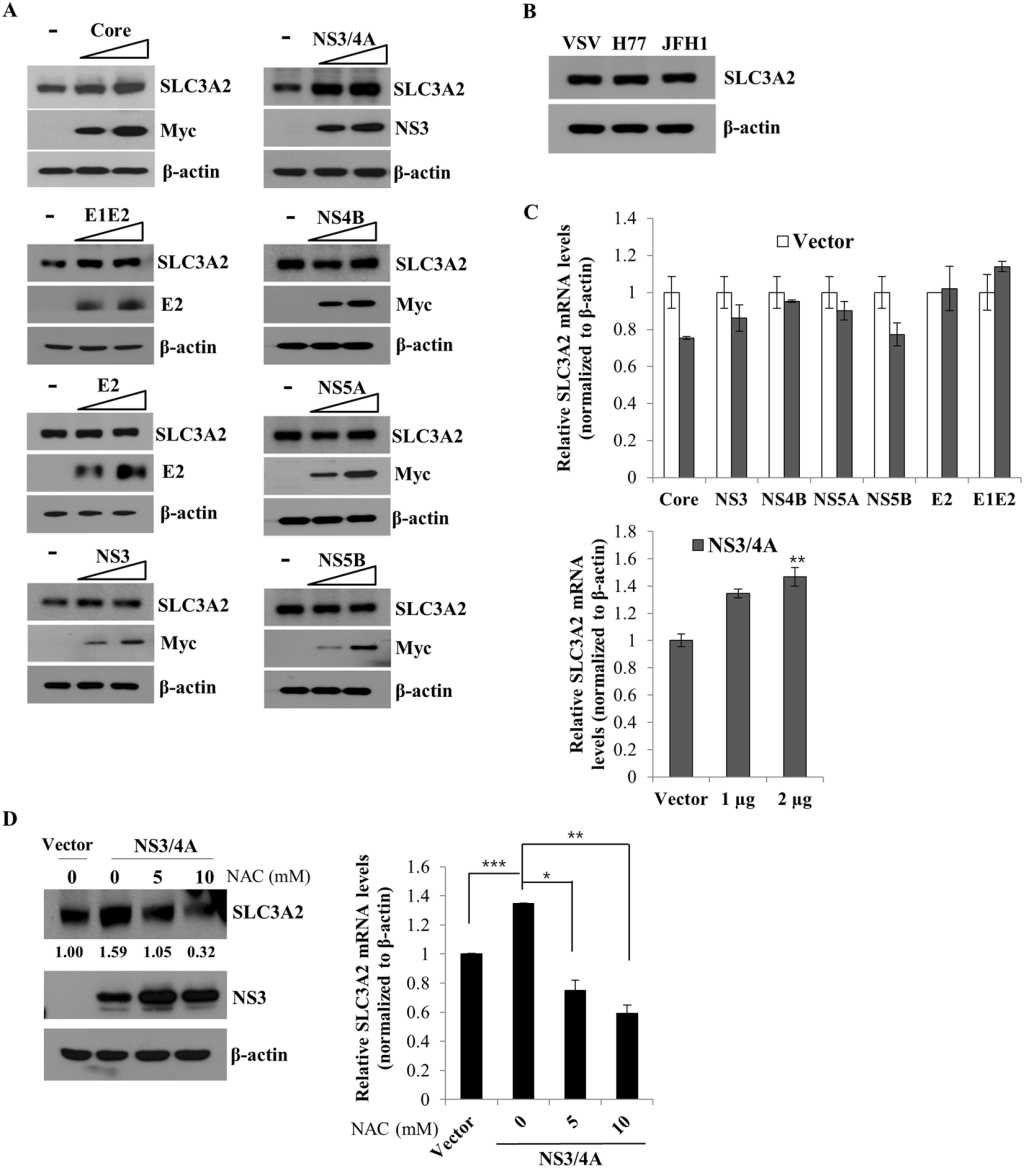
SLC3A2 expression is upregulated by NS3/4A-induced oxidative stress. (**A**) Huh7.5 cells were transiently transfected with either vector (−) or increasing amounts of Myc-tagged core, NS3, NS3/4A, NS4B, NS5A, NS5B, or V5-tagged E2, E1E2 expression plasmid, respectively. At 36 h after transfection, total cell lysates were immunoblotted with the indicated antibodies. (**B**) Huh7.5 cells were infected with either VSVpp harboring HCV envelope protein or HCVpp harboring HCV envelope protein derived from H77 or JFH1 for 6 h. At 48 h postinfection, SLC3A2 protein expression levels were analyzed by immunoblot analysis. (**C**) (Upper panel) Huh7.5 cells were transiently transfected with empty vector, Myc-tagged core, NS3, NS4B, NS5A, NS5B, or V5-tagged E2, E1E2 expression plasmid, respectively. At 36 h after transfection, mRNA levels of SLC3A2 were determined by qRT-PCR analysis. (Lower panel) Huh7.5 cells were transiently transfected with either empty vector or increasing amounts of NS3/4A expression plasmid. At 36 h after transfection, mRNA levels of SLC3A2 were determined by qRT-PCR analysis. (**D**) Huh7.5 cells were transfected with either vector or NS3/4A expression plasmid. At 36 h after transfection, cells were treated with increasing amount of NAC. At 24 h after treatment, both protein (left panel) and mRNA levels (right panel) were analyzed by either immunoblot assay or qRT-PCR, respectively.

Since SLC3A2 expression levels are increased in both HCV infected cells and HCV replicon cells, nonstructural proteins may be responsible for this upregulation. To explore this hypothesis, Huh7.5 cells were transfected with either vector or increasing amounts of individual HCV protein expression plasmid and then SLC3A2 protein expression was analyzed by immunoblot analysis. As shown in Fig. 3A, NS3/4A markedly increased SLC3A2 protein expression level. Since SLC3A2 protein expression level was also minimally increased by E1E2, we further explored the possible involvement of E1E2 on SLC3A2 expression by infecting Huh7.5 cells with either VSVpp or HCVpp derived from either H77 or JFH1. Figure 3B shows that SLC3A2 protein expression was not upregulated by E1E2, indicating that HCV NS3/4A specifically upregulated SLC3A2 protein level. Consistently, mRNA level of SLC3A2 was also significantly increased by NS3/4A but not by other HCV proteins (Fig. 3C). It has been previously reported that NS3/4A showed an approximately 11-fold increase in ROS level in Huh7.5.1 cells^18^. To investigate whether SLC3A2 upregulation was modulated by NS3/4A-induced ROS, Huh7.5 cells transfected with NS3/4A plasmid were treated with NAC. As shown in Fig. 3D, protein level of SLC3A2 was markedly increased by NS3/4A. However, NS3/4A-induced SLC3A2 protein level was decreased to basal level by NAC in a dose-dependent manner (left panel). Consistently, mRNA level of SLC3A2 was significantly reduced by NAC (Fig. 3D, right panel), indicating that NS3/4 A played a crucial role in upregulation of SLC3A2.

### SLC3A2/LAT1 complex level was increased in the context of HCV infection

**Figure 4:**
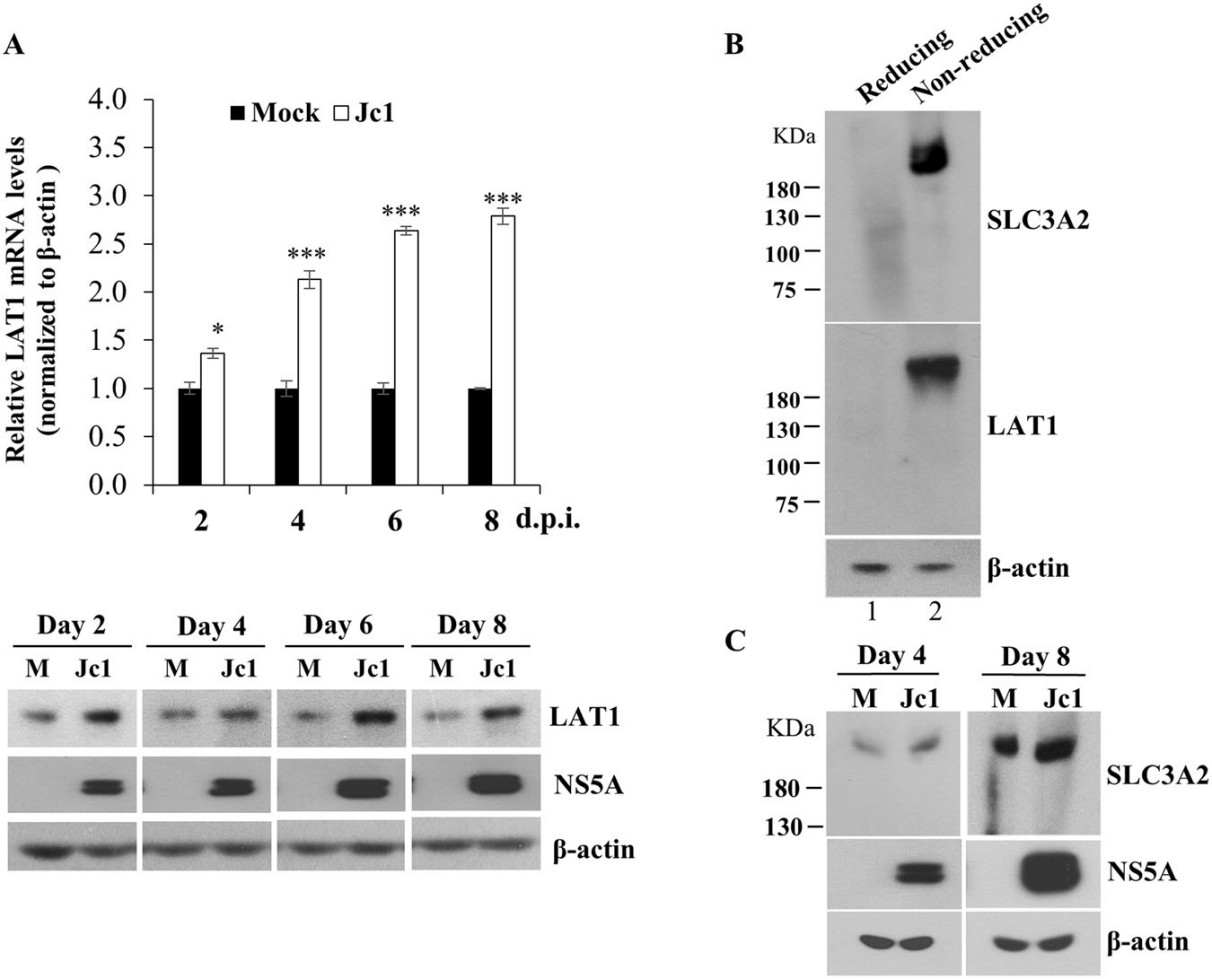
SLC3A2/LAT1 complex is upregulated by HCV infection. (**A**) Huh7.5 cells were either mock-infected or infected with Jc1 for the indicated time intervals. Both mRNA (top panel) and protein (bottom panel) levels of LAT1 were analyzed either by qRT-PCR or immunoblot analysis, respectively. (**B**) Huh7.5 cells were treated with either reducing or non-reducing buffer as described in Methods. Total cell lysates were subjected to native gel electrophoresis and immunoblotted with either SLC3A2 or LAT1 antibodies, respectively. (**C**) Huh7.5 cells were either mock-infected or infected with Jc1 for 4 and 8 days, respectively. Total cell lysates prepared by native lysis buffer were immunoblotted with the indicated antibodies.

SLC3A2 (CD98) and LAT1 (SLC7A5) constitute a heterodimeric transmembrane protein complex that catalyzes amino acid transport^19^. We therefore wondered whether LAT1 expression level was also upregulated by HCV infection. For this purpose, Huh7.5 cells were either mock-infected or infected with Jc1 and then both mRNA and protein levels of LAT1 were determined. Figure 4A shows that mRNA levels of LAT1 were gradually increased during Jc1 infection (upper panel). Consistently, protein levels of LAT1 were markedly increased in the context of HCV infection (Fig. 4A, lower panel). We next investigated whether SLC3A2/LAT1 heterodimeric complex level was also modulated by HCV infection. We first examined whether SLC3A2 formed a heterodimeric complex with LAT1 in hepatic cells. For this purpose, Huh7.5 cells treated with either reducing or non-reducing buffer were subjected to native gel electrophoresis and analyzed by immunoblot analysis. As shown in Fig. 4B, the same size of heterodimeric complex was detected by either anti-SLC3A2 or anti-LAT1 antibodies in cells treated with non-reducing buffer (lane 2). However, this complex was not detectable under reducing condition (Fig. 4B, lane 1), suggesting that both SLC3A2 and LAT1 formed a heterodimeric complex via disulfide bonds. To determine how HCV modulated SLC3A2/LAT1 heterodimeric complex level, Huh7.5 cells were either mock-infected or infected with Jc1 and then total cell lysates harvested at the indicated time points were analyzed under non-reducing condition. Figure 4C shows that protein mass corresponding to the SLC3A2/LAT1 heterodimeric complex was detected in both mock- and Jc1-infected cells. Of note, SLC3A2/LAT1 complex level was markedly increased in the context of HCV infection.

### HCV modulates SLC3A2/LAT1 complex to promote cell proliferation

**Figure 5:**
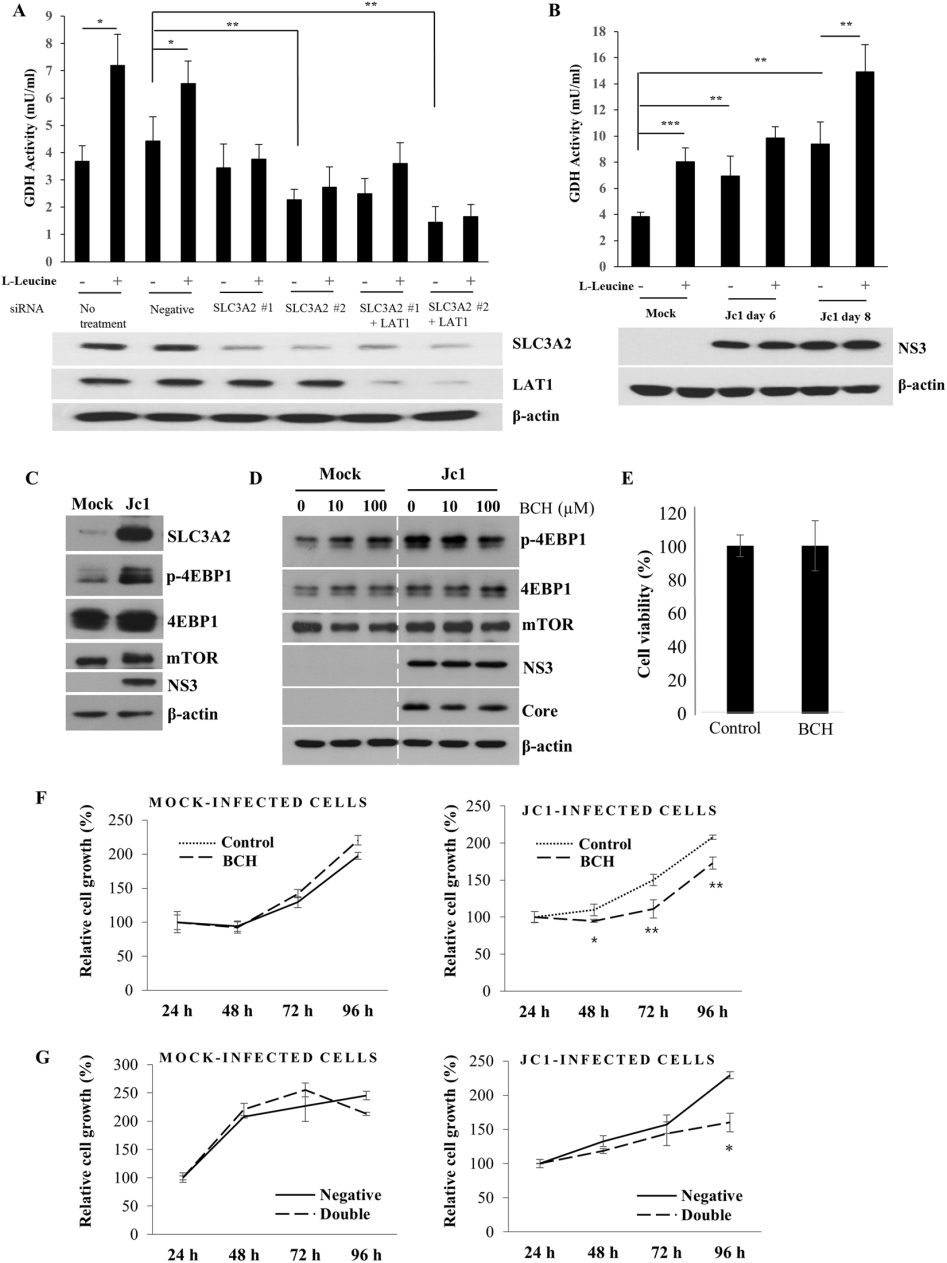
HCV modulates SLC3A2/LAT1 complex to promote cell proliferation. (**A**) (Upper panel) Huh7.5 cells were transfected with negative siRNA, SLC3A2-specific siRNA or cotransfected with SLC3A2 and LAT1 siRNAs. At 48 h after transfection, cells were either left untreated or treated with 5 mM L-leucine. At 4 h after treatment, GDH activity was measured according to the manufacturer’s instructions. (Lower panel) Cell lysates harvested at 48 after siRNA transfection were immunoblotted with the indicated antibodies. (**B**) (Upper panel) Huh7.5 cells were either mock-infected or infected with Jc1 for 4 h. At the indicated time points, cells were either left untreated or treated with 5 mM L-leucine. At 4 h after treatment, GDH activity was measured according to the manufacturer’s instructions. (Lower panel) Cell lysates harvested at the indicated time points were immunoblotted with the indicated antibodies. (**C**) Huh7.5 cells were either mock-infected or infected with Jc1. At 6 days postinfection, total cell lysates were immunoblotted with the indicated antibodies. (**D**) Huh7.5 cells infected with either mock or Jc1 for 6 days were treated with BCH for 2 h. Total cell lysates were immunoblotted with the indicated antibodies. (**E**) Huh7.5 cells were treated with either control vehicle (NH_4_OH) or 200 µM of BCH. At 96 h after treatment, cell viability was determined by WST assay. (**F**) Huh7.5 cells infected with either mock (left panel) or Jc1 (right panel) for 4 days were treated with either control vehicle (NH_4_OH) or BCH. At the indicated time points, cell proliferation was analyzed by WST assay. (**G**) Huh7.5 cells were either mock infected (left panel) or infected with Jc1 (right panel). At 4 days postinfection, cells were transfected with either negative siRNA or siRNA mix targeting both SLC3A2 and LAT1 (double). At the indicated time points, cell proliferation was assessed by WST assay.

It has been previously reported that HCV activates the mTORC1 signaling^20^, and SLC3A2/LAT1 heterodimeric amino acid transporter regulates mTORC1 signaling through transporting leucine into the cells^7,8^. To investigate the involvement of SLC3A2/LAT1 on amino acid transport, Huh7.5 cells treated with the indicated siRNAs were either left untreated or treated with L-leucine and then glutamate dehydrogenase (GDH) activity was determined. Because L-leucine stimulates GDH, L-leucine transport is measured by GDH activity. Figure 5A showed that GDH activity was significantly increased in the presence of L-leucine. Of note, L-leucine-induced GDH activity was significantly decreased in both SLC3A2-silenced cells and SLC3A2/LAT1-knockdown cells. To further investigate whether amino acid level is upregulated by HCV infection, Huh7.5 cells infected with either mock or Jc1 were treated with L-leucine and GDH activity was determined. We showed that GDH activity was increased with L-leucine treatment in mock-infected cells (Fig. 5B). Importantly, GDH activity was significantly increased in Jc1-infected cells and Jc1-induced GDH activity was further enhanced in the presence of L-leucine. These data indicate that HCV increases SLC3A2/LAT1 complex level to facilitate amino acid transport in HCV-infected cells. In addition, the mTORC1 pathway known to regulate cell proliferation is mainly mediated through eukaryotic initiation factor 4E (eIF4E) binding protein 1 (4EBP1)^21,22^. We showed that phosphorylation level of 4EBP1 was markedly increased in HCV-infected cells as compared with mock-infected cells (Fig. 5C). These data indicated that 4EBP1, downstream target of mTORC1, was activated in HCV infected cells, suggesting that mTORC1 signaling was upregulated by SLC3A2/LAT1 heterodimeric amino acid transporter. To further corroborate this result, Huh7.5 cells infected with either mock or Jc1 were treated with BCH, an inhibitor of L-Leucine transporter, and then total cell lysates were immunoblotted with the indicated antibodies. Figure 5D shows that HCV-induced 4EBP1 phosphorylation level was gradually decreased with increasing amounts of BCH. We further showed that treatment of the same concentration of BCH displayed no effect on cell viability, demonstrating that the effect of BCH was specific to L-Leucine transporter (Fig. 5E). We further evaluated the role of SLC3A2/LAT1 complex in cell proliferation. Huh7.5 cells infected with either mock or Jc1 were treated with either vehicle (NH_4_OH) or BCH and then cell proliferation was analyzed by WST assay. As shown in Fig. 5F, BCH significantly decreased cell growth in HCV-infected cells as compared with mock-infected cells, suggesting that leucine uptake by SLC3A2/LAT1 transporter was required for cell proliferation. To further confirm this result, Huh7.5 cells infected with either mock or Jc1 were transfected with either negative siRNA or siRNAs targeting both SLC3A2 and LAT1 (double). Figure 5G verified that cell growth in HCV-infected cells was significantly decreased in SLC3A2 and LAT1 knockdown cells as compared with mock-infected cells. Collectively, these data suggest that HCV modulates SLC3A2/ LAT1 complex through mTORC1 signaling pathway to promote cell proliferation.

### SLC3A2 is required for HCV propagation

**Figure 6:**
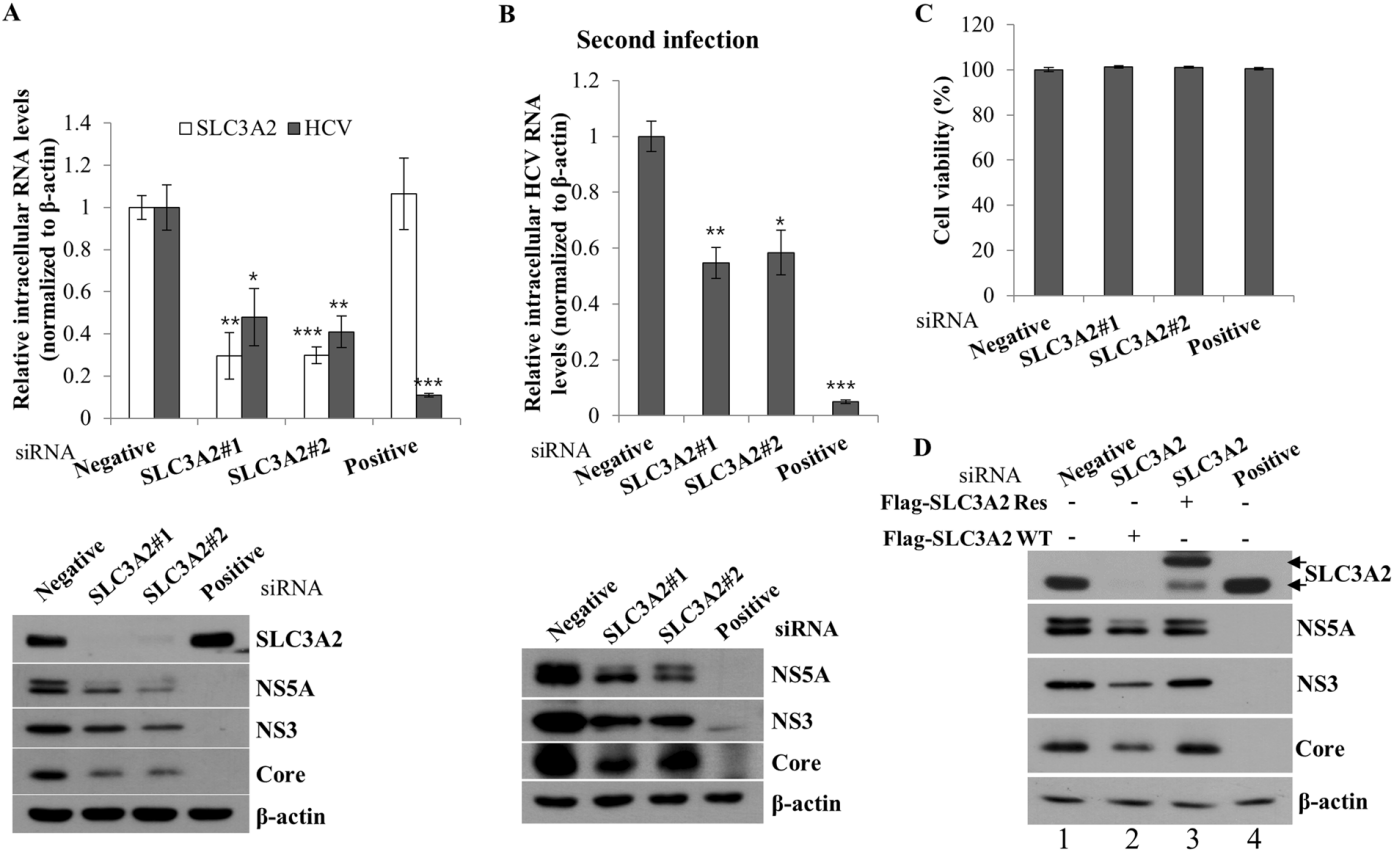
SLC3A2 is required for HCV propagation. (**A**) Huh7.5 cells were transfected with 40 nM of the indicated siRNAs for 48 h and then infected with Jc1. At 48 h postinfection, intracellular RNA levels were analyzed by qRT-PCR (upper panel), and protein levels were determined by immunoblot assay using the indicated antibodies (lower panel). (**B**) Naïve Huh7.5 cells were infected with Jc1 in supernatant harvested from the first infection. At 48 h postinfection, both RNA (upper panel) and protein (lower panel) levels were determined by qRT-PCR and immunoblot analysis, respectively. (**C**) Huh7.5 cells were transfected with 40 nM of the indicated siRNAs, individually. At 96 h after transfection, cell viability was assessed by WST assay. Negative, universal negative control siRNA; positive, HCV-specific siRNA targeting the 5′ nontranslated region (NTR) of Jc1. (**D**) Huh7.5 cells were transfected with the indicated siRNAs. At 24 h after transfection, cells were further transfected with either wild-type or siRNA-resistant SLC3A2 mutant plasmid, followed by Jc1 infection. At 48 h postinfection, total cell lysates were immunoblotted with the indicated antibodies. Flag-SLC3A2 WT, Flag-tagged SLC3A2 wild-type; Flag-SLC3A2 Res, Flag-tagged siRNA-resistant SLC3A2 mutant.

To explore the possible involvement of SLC3A2 in HCV propagation, Huh7.5 cells transfected with either negative control siRNA or SLC3A2-specific siRNA were infected with Jc1, and then both mRNA and protein levels were determined. Silencing of SLC3A2 significantly suppressed RNA expression level of HCV (Fig. 6A, upper panel). Consistently, protein expression levels of HCV were impaired in SLC3A2 knockdown cells (Fig. 6A, lower panel). To further investigate the role of SLC3A2 in HCV propagation, naïve Huh7.5 cells were infected with Jc1 in supernatant harvested from the primary infection, and then both RNA and protein levels of HCV were analyzed. As shown in Fig. 6B, viral infectivity determined by both viral RNA and protein levels in the second infection was markedly decreased by knockdown of SLC3A2. Moreover, treatment of the same concentration of siRNAs displayed no cytotoxicity in the cells, indicating that the silencing effect was specific to SLC3A2 (Fig. 6C). To rule out the off-target effect of a SLC3A2 siRNA, we generated an siRNA-resistant SLC3A2 mutant. Figure 6D showed that exogenous expression of the siRNA-resistant SLC3A2 mutant, but not of the wild-type SLC3A2, restored the HCV protein expression levels (lane 3 versus lane 4). Collectively, these data suggest that SLC3A2 is specifically required for HCV propagation.

### SLC3A2 is not involved in the replication, translation, and virion production steps of the HCV life cycle

**Figure 7:**
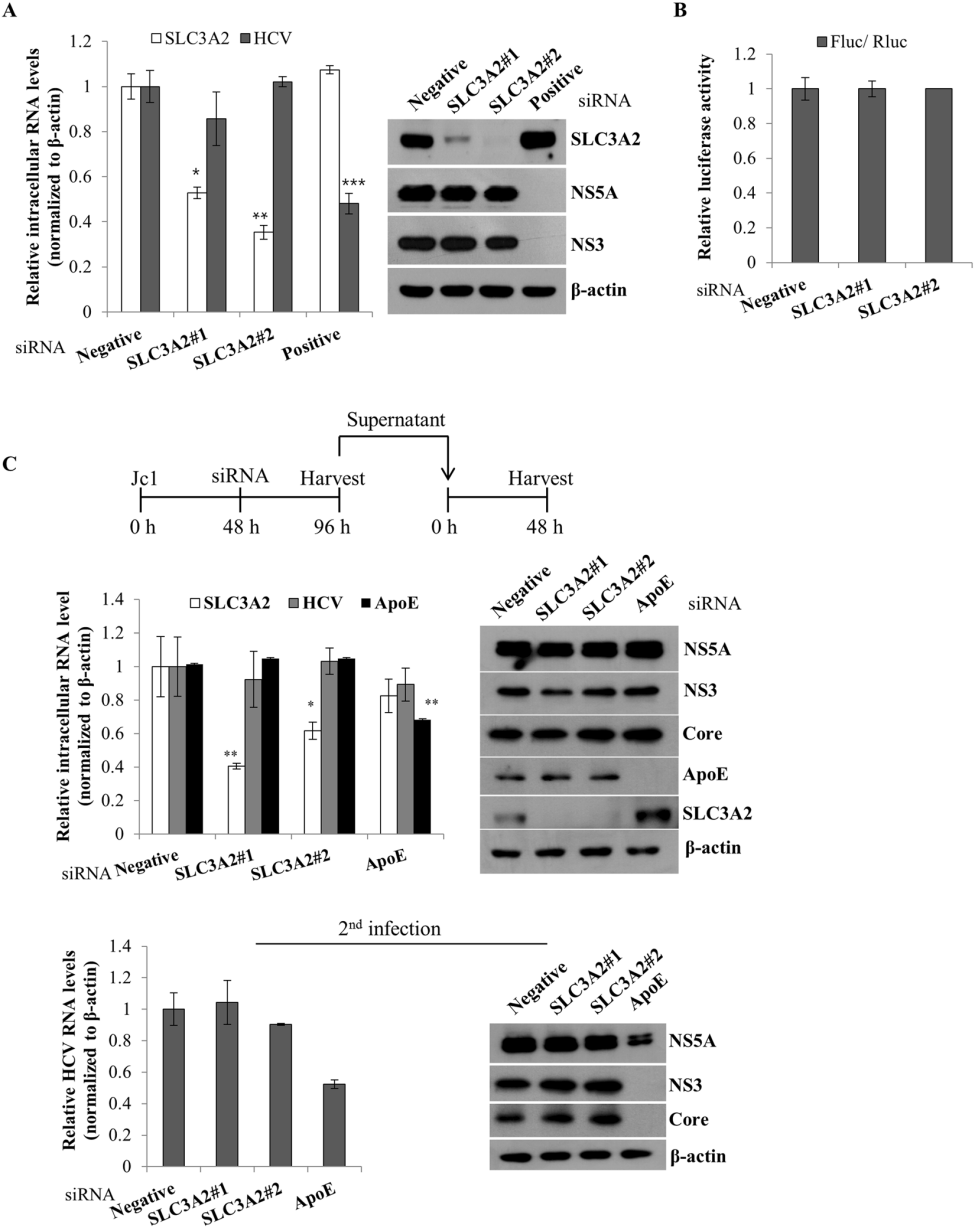
SLC3A2 is not involved in the replication, translation, and assembly/release steps of the HCV life cycle. (**A**) Huh7 cells harboring HCV subgenomic replicon derived from genotype 1b were transfected with the indicated siRNAs. At 72 h after siRNA transfection, both RNA (left) and protein (right) levels were analyzed by qRT-PCR and immunoblot assay, respectively. (**B**) Huh7.5 cells were transfected with the indicated siRNAs. At 48 h after siRNA transfection, cells were further transfected with pRL-HL dual luciferase and a pCH110 β-galactosidase plasmid. At 48 h after transfection, relative luciferase activity was determined. (**C**) (Top panel) A schematic illustration of the experimental design. (Middle panels) Huh7.5 cells were infected with Jc1 for 48 h and then transfected with 40 nM of the indicated siRNAs. At 48 h after transfection, both intracellular RNA (left) and protein (right) levels were determined by qRT-PCR and immunoblot assay, respectively. (Bottom panels) Naïve Huh7.5 cells were infected with Jc1 in supernatant harvested from the first infection. At 48 h postinfection, both RNA (left) and protein (right) levels were determined by qRT-PCR and immunoblot assay, respectively.

To investigate which step of the HCV life cycle was required for SLC3A2, we analyzed the SLC3A2 expression level in Huh7 cells harboring HCV subgenomic replicon by silencing of SLC3A2. As shown in Fig. 7A, knockdown of SLC3A2 displayed no effect on intracellular HCV RNA (left panel) and protein (right panel) levels in replicon cells derived from genotype 1b. We next investigated whether SLC3A2 was involved in HCV internal ribosome entry site (IRES)-mediated translation. For this purpose, Huh7.5 cells were transiently transfected with the indicated siRNAs together with pRL-HL and a β-galactosidase plasmid, and then luciferase activity was determined as we reported previously^12^. Figure 7B demonstrated that silencing of SLC3A2 exerted no effects on HCV IRES-mediated translation. To examine if SLC3A2 was required for a later stage of the HCV life cycle, Huh7.5 cells were first infected with Jc1 for 48 h and then further transfected with either a negative siRNA or a siRNA targeting SLC3A2 or an apolipoprotein E (ApoE) siRNA as a control. At 48 h after transfection, both RNA and protein levels were determined. Figure 7C shows that silencing of SLC3A2 displayed no effect on both RNA and protein levels of HCV (upper panel). To verify this result, naïve Huh7.5 cells were infected with HCV derived from culture supernatant harvested from the first infection. As shown in Fig. 7C (lower panels), silencing of SLC3A2 had no discernible effect on viral infectivity. All these data indicated that SLC3A2 was not involved in the replication, translation, and virion production steps of the HCV life cycle.

### SLC3A2 is required in the entry step of the HCV life cycle

**Figure 8:**
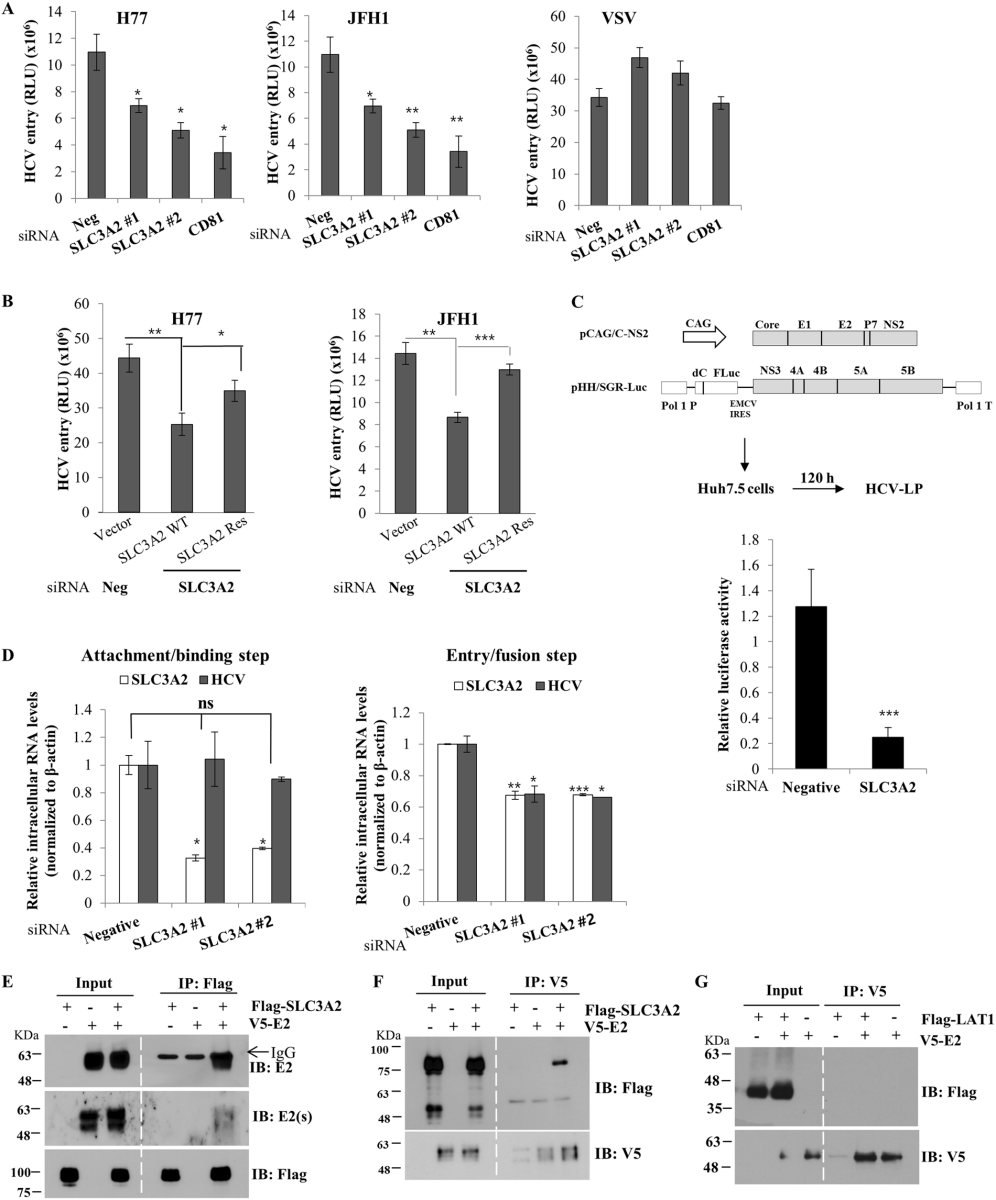
SLC3A2 is required for the entry step of the HCV life cycle. (**A**) Huh7.5 cells were transfected with the indicated siRNAs. At 48 h after siRNA transfection, cells infected with HCVpp derived from either genotype 1b (H77) or genotype 2a (JFH1) for 6 h. VSVpp was used as a control. Cell medium was replaced with fresh DMEM containing antibiotics. At 48 h postinfection, cells were harvested and then luciferase activity was determined. (**B**) Huh7.5 cells were transfected with either negative or SLC3A2-specific siRNA for 24 h, followed by transfection with Flag-tagged siRNA-resistant SLC3A2 mutant plasmid. At 24 h after plasmid transfection, cells were infected with HCVpp derived from either H77 (left) or JFH1 (right). At 48 h postinfection, total cells were harvested and luciferase activities were determined. (**C**) (Left panel) Schematic diagram of the plasmids used for the production of HCV-LP. (Right panel) Huh7.5 cells were transfected with either negative or SLC3A2-specific siRNAs. At 48 h after siRNA transfection, cells were infected with HCV-LP. At 48 h postinfection, total cells were harvested and luciferase activities were determined for single cycle infection. Data represent averages from at least three independent experiments. The asterisks indicate significant differences (***p < 0.001) from the activity for the negative control. (**D**) (Left panel) Huh7.5 cells were transfected with the indicated siRNAs for 48 h. The cells were incubated with Jc1 at 4 °C for 2 h for binding. The cells were washed with PBS and then bound virions were determined by analyzing HCV RNA levels by qRT-PCR. (Right panel) Huh7.5 cells were transfected with the indicated siRNAs. At 48 h after transfection, cells were incubated with Jc1 at 4 °C for 2 h. The cells were washed with PBS and then temperature was shifted to 37 °C for 4 h. The cells were trypsinized and washed twice with PBS to remove free virions. Internalized HCV virions were indirectly determined by analyzing HCV RNA levels by qRT-PCR. (**E**) HEK293T cells were cotransfected with Flag-tagged SLC3A2 and either vector or V5-tagged E2 expression plasmid. At 36 h after transfection, total cell lysates were immunoprecipitated with an anti-Flag antibody and bound proteins were analyzed by immunoblot analysis using an anti-E2 antibody and secondary antibodies. To verify E2 protein, we used another kind of secondary antibody, E2(s), which did not recognize heavy chain. (**F**) Total cell lysates prepared as described in (E) were immunoprecipitated with anti-V5 antibody and bound proteins were detected by immunoblot analysis using an anti-Flag antibody. (**G**) HEK293T cells were cotransfected with Flag-tagged LAT1 and either vector or V5-tagged E2 plasmid. At 30 h after transfection, total cell lysates were immunoprecipitated with an anti-V5 antibody and bound proteins were analyzed by immunoblot analysis using an anti-Flag antibody.

Since SLC3A2 was not involved in replication and production steps, one remaining possibility was that SLC3A2 might play a role in the entry step of the HCV life cycle. To explore this possibility, we performed viral entry assays using HCVpp derived from either H77 or JFH1. The vesicular stomatitis glycoprotein G (VSVpp) was used as a control. As shown in Fig. 8A, HCVpp entry was impaired in SLC3A2 knockdown cells in both genotypes. However, VSVpp entry was not altered by knockdown of SLC3A2. To further verify the involvement of SLC3A2 in the HCV entry, Huh7.5 cells transfected with either negative or SLC3A2-specific siRNA were further transfected with either Flag-tagged wild-type SLC3A2 or siRNA-resistant SLC3A2 mutant plasmid. At 24 h after transfection, cells were infected with HCVpp derived from either H77 or JFH1. As shown in Fig. 8B, HCV entry was recovered by siRNA-resistant SLC3A2 mutant, but not by wild-type SLC3A2, indicating that SLC3A2 was specifically required for HCV entry. To further confirm this result, we performed viral entry assays using HCV like particle (HCV-LP) as we described previously^23^. In this system, HCV-LP can undergo single cycle infection without producing virions. We produced HCV-LP as depicted in Fig. 8C (left panel). Huh7.5 cells were transfected with either negative siRNA or siRNA targeting SLC3A2 and then infected with HCV-LP for 48 h. As shown in Fig. 8C (right panel), HCV-LP luciferase activity was significantly reduced in SLC3A2 knockdown cells as compared to the negative control. To further investigate the precise involvement of SLC3A2 in HCV entry, we divided HCV infection steps into attachment/binding and entry/fusion steps. To analyze if SLC3A2 was required during attachment/binding step, Huh7.5 cells were transfected with either negative control siRNA or SLC3A2-specific siRNA for 48 h and then incubated with Jc1 inoculum for 2 h at 4 °C. After washing with PBS, bound virions were determined by HCV RNA levels. As shown in Fig. 8D, silencing of SLC3A2 displayed no effect on HCV binding to the host cells (left panel). To determine if SLC3A2 was required during entry/fusion step, Huh7.5 cells transfected with either negative control siRNA or SLC3A2-specific siRNA for 48 h were incubated with Jc1 inoculum for 2 h at 4 °C and washed with PBS to remove unbound virions. Temperature was then shifted to 37 °C for 4 h to allow virions to internalize the host cells. The cells were trypsinized and non-internalized virions were washed away with PBS. Virion entry was determined by analyzing intracellular HCV RNA levels. Figure 8D demonstrated that HCV RNA level was significantly decreased in SLC3A2 knockdown cells (right panel), indicating that SLC3A2 is a host factor required at a post-binding step in HCV entry. Since SLC3A2 regulated HCV entry and mTORC1 signaling in complex with LAT1, we postulated that HCV envelope protein might be associated with SLC3A2. For this purpose, HEK293T cells were cotransfected with Flag-tagged SLC3A2 and either vector or V5-tagged E2 expression plasmid. Total cell lysates harvested at 36 h after transfection were immunoprecipitated with an anti-Flag antibody and bound protein was detected by an anti-E2 antibody. We showed that SLC3A2 interacted with HCV E2 protein (Fig. 8E). To verify this result, the same cell lysates were immunoprecipitated with an anti-V5 antibody and bound protein was analyzed by immunoblot assay using an anti-Flag antibody. Reciprocal coimmunoprecipitation experiment confirmed that E2 interacted with SLC3A2 (Fig. 8F). However, LAT1 did not interact with HCV E2 (Fig. 8G), further confirming that SLC3A2 specifically interacted with E2 protein. Collectively, these data suggest that HCV may coopt host SLC3A2 protein via E2 to facilitate viral entry.

## Discussion

SLC3A2 (also known as CD98 heavy chain, CD98hc) and large neutral amino acid transporter 1 (LAT1) form a heterodimer and interact with integrins. SLC3A2 regulates the expression and distribution of the light chains to modulate amino acid transport, and overexpression of SLC3A2 promotes tumorigenesis^6^. Indeed, SLC3A2 was overexpressed in many cancer cell lines, including lung, colon, and breast^24^, and it is also known as a novel marker for renal cancer^25^. Our recent proteomic analysis data show that SLC3A2 is highly expressed in HCVcc-infected cells^12^. In the present study, we for the first time showed that both mRNA and protein expression levels of SLC3A2 were upregulated in the context of HCV replication. We also demonstrated that ROS significantly increased SLC3A2 expression levels. It has been previously reported that HCV induces ROS production in hepatoma cells^16^. Using NAC, we verified that the upregulation of SLC3A2 expression in HCV-infected cells was mediated by oxidative stress. Since core, NS3/4A, and NS5A proteins are involved in ROS production^14–16^, why NS3/4A but not core and NS5A upregulate SLC3A2 expression needs further studies. We tempted to speculate that protease enzymatic activity of NS3/4A together with ROS may be involved in SLC3A2 upregulation by unknown mechanism.

Since NS3/4A-induced mRNA and protein levels of SLC3A2 are markedly decreased by NAC (Fig. 3D), HCV upregulates SLC3A2 expression level via NS3/4A-mediated oxidative stress.

SLC3A2 forms a complex with LAT1 for amino acid transport activity^4,5,7^. We demonstrated that LAT1 expression level was gradually increased during HCV infection and SLC3A2/LAT1 complex level was also elevated in Jc1-infected cells. SLC3A2/LAT1 complex plays a critical role in the uptake of extracellular leucine, which subsequently leads to mTORC1 activation^24,26^. By siRNA-mediated silencing of SLC3A2/LAT1, we verified that SLC3A2/LAT1 complex was involved in L-leucine transport. Importantly, L-leucine transport level was significantly increased in Jc1-infected cells as compared with mock-infected cells. All these data indicate that the increase in amino acid transport in HCV-infected cells may be due to enhanced SLC3A2/LAT1 expression level. We then investigated the phosphorylation level of 4EBP1, a downstream target protein of mTORC1, in HCV infected cells. As expected, phospho-4EBP1 level was remarkably increased by HCV infection and HCV-induced phospho-4EBP1 level was pronouncedly decreased by BCH, indicating that SLC3A2/LAT1 complex was upregulated by HCV. Furthermore, cell proliferation was significantly decreased in both BCH-treated and SLC3A2/LAT1 knockdown cells, suggesting that HCV promoted cell proliferation by increasing leucine transport via mTORC1 signaling pathway for persistent infection.

Oxidative stress is an event of enhanced formation of ROS in the cells^27^. ROS plays an important role in determining the progression of HCV-induced liver pathogenesis^28^. Oxidative stress in HCV-infected cells occurs by a combination of chronic inflammation and proteins encoded by HCV. HCV core, NS3/4A, and NS5A proteins primarily cause an increase in ROS production^14–16^. In addition to ROS production, the mTORC1 can be also activated by HCV infection^17,18^. It has been previously reported that HCV NS5A activates mTOR signaling pathway to promote persistent infection^29^. mTORC1 consists of mTOR, raptor, mLST8, PRAS40, and DEPTOR. mTOR, core component of mTORC1/2, is a serine/threonine kinase that is involved in the regulation of multiple cellular processes, including cell growth, proliferation, protein synthesis, and survival. mTORC1 is activated by amino acids such as leucine and hormones, including leptin, insulin, and IGF-1^20^. However, the mechanism by which HCV hijacks mTORC1 signaling pathway has not been fully understood.

We then assessed the functional role of SLC3A2 in HCV propagation. Knockdown of SLC3A2 impaired both RNA and protein expressions of HCV, implying that SLC3A2 positively regulated HCV propagation. Using an HCVpp entry assay, we showed that HCV entry was impaired in SLC3A2 knockdown cells. By analyzing the precise involvement of SLC3A2 in HCV infection, we further showed that SLC3A2 was specifically required for post-binding step in the HCV entry. This result suggests that SLC3A2 may be a novel host cofactor involved in HCV entry. HCV entry is a complex process that requires multiple cell surface factors, including CD81, scavenger receptor class B type I (SR-BI), claudin 1 (CLDN1), occludin (OCLN), and Niemann-Pick C1-like 1 cholesterol absorption receptor (NPC1L1)^30–34^. Furthermore, HCV internalizes the host cells via clathrin-mediated endocytosis^35^. We also demonstrated that SLC3A2 interacted with HCV E2 protein. It has been previously reported that HCV E2 interacts with SR-BI at post-binding step to enhance viral infectivity^33^. Moreover, CD81 interacts with HCV E2 via a series of discontinuous amino acid residues in the large extracellular loop^36^ and CD81 acts as a post-binding entry molecule^37^. Likewise, SLC3A2 may interact with E2 to facilitate HCV entry into the host cells. Interestingly, SLC3A2 has been also implicated in vaccinia virus entry^38^. Although SLC3A2 is identified as an additional cofactor involved in HCV entry, how SLC3A2 functions in conjunction with those already known cofactors in HCV entry needs further studies. In summary, HCV upregulated SLC3A2 expression levels via NS3/4A-mediated oxidative stress. Furthermore, HCV increased the SLC3A2/LAT1 heterodimeric amino acid transporter level to promote viral propagation. Our study provides some compelling evidence showing that SLC3A2 is an essential cellular cofactor required for HCV entry and thus SLC3A2 could be a potential therapeutic target to interrupt HCV propagation.

## Methods

### Plasmid constructions and DNA transfection

Total cellular RNAs were isolated from Huh7 cells by using RiboEx (GeneAll), and full-length SLC3A2 was amplified by a primer set (sense, 5′-AAG CGG CCG CTA TGG AGC TAC AGC CT-3′; antisense, 5′-AAG GTA CCT CAG GCC GCG TAG GGG AA-3′) from cDNA synthesized by using a cDNA synthesis kit (Toyobo) according to the manufacturer’s instructions. PCR products were inserted into the BamH1 and Xba1 sites of plasmid pEF-V5-His (Invitrogen) to generate V5-tagged SLC3A2, and the Not1 and Kpn1 sites of the p3xFlag-CMV10 vector (Sigma-Aldrich) to generate Flag-tagged SLC3A2 expression plasmid. Myc-tagged HCV core, NS3, NS4B, NS5A, and NS5B, V5-tagged HCV E2, E1E2, NS3/4A plasmids were described previously^12,39^. All DNA transfections were performed by using polyethyleneimine (Sigma-Aldrich) as we described previously^12^.

### Cell culture

All cell lines were grown in Dulbecco’s modified Eagle’s medium (DMEM) supplemented with 10% fetal calf serum, 1% penicillin-streptomycin, and 1% non-essential amino acids in 5% CO_2_ at 37 °C. Huh7 cells harboring HCV subgenomic replicon derived from genotype 1b were grown as described elsewhere^12^.

### Antibodies and chemicals

Antibodies were purchased from the following sources: SLC3A2 (catalogue #2671), mTOR (#2983), phospho-mTOR (#5536), 4EBP1 (#9644), and phospho-4EBP1 (#2855) antibodies were from Cell Signaling, Flag (#SLBQ 7119V) and β-actin (#103M4784V) antibodies were from Sigma-Aldrich, LAT1 (sc-53550) and Myc (#D0618) antibodies were from Santa Cruz, V5 (#1865511) antibody was from Invitrogen. HCV core, NS3, NS5A, and NS5B antibodies have been described elsewhere^23^. HCV E2 antibody was a gift from Dr. Jean Dubuisson (Institut Pasteur de Lille). N-acetyl-cysteine (NAC) (#616-91-1), an antioxidant agent, and 2-aminobicyclo [2.2.1] heptane-2-carboxylic acid (BCH) (#20448-79-7), L-leucine transporter inhibitor, were purchased from Sigma-Aldrich. BAPTA-AM (BML-CA411-0025), an intracellular Ca2+ chelator, was purchased from Enzo.

### Immunoblot analysis

Cells were washed twice with phosphate-buffered saline (PBS) and lysed in either radioimmunoprecipitation assay (RIPA) buffer (20 mM Tris HCl [pH 7.4], 150 mM NaCl, 10% glycerol, 1% NP-40, 10 mM NaF, 30 mM sodium pyrophosphate, 1 mM EDTA, 1 mM Na_3_VO_4_, 1 mM PMSF, and protease inhibitor cocktail) for reducing samples or native lysis buffer (50 mM Tris–HCl, 150 mM NaCl, 0.5 mM EDTA and protease inhibitors) for non-reducing samples for 15 min on ice and centrifuged at 13,000 rpm for 10 min at 4 °C. The supernatant was collected and protein concentration was determined by the Bradford assay kit (Bio-Rad). Equal amounts of proteins were subjected to either SDS-PAGE or non-denaturing native PAGE, and electro-transferred to a PVDF membrane. The membrane was blocked in Tris-buffered saline (TBS)-Tween (20 mM Tris-HCl [pH 7.6], 150 mM NaCl, and 0.25% Tween 20) containing 5% nonfat dry milk for 1 h, and then incubated with the indicated primary and secondary antibodies, respectively. Proteins were detected using an ECL kit (Abfrontier).

### RNA interference

siRNAs targeting SLC3A2 (SLC3A2 siRNA #1, 5′-CUC AAC UUC UCC GAC UCU A-3′ and SLC3A2 siRNA #2, 5′-CAG AUC CUG AGC CUA CUC GAA-3′), LAT1 (5′-CUC UUU GCC UAU GGA GGA U-3′) and the universal negative control siRNA were purchased from Bioneer (South Korea). An siRNA targeting the 5′ nontranslated region (NTR) of the HCV Jc1 virus (5′-CCU CAA AGA AAA ACC AAA CUU-3′) was used as a positive control^12^. Jc1 is the most efficient GT2a/2a chimera construct consisting of J6CF- and JFH1-derived sequences that yields infectious titers 100- to 1,000-fold higher than the parental isolate and all other chimeras^13^. siRNA transfection was performed using Lipofectamine RNAiMax reagent (Invitrogen, Carlsbad, CA) according to the manufacturer’s instructions.

### WST assay

Cells seeded on 48-well plates were either transfected with the indicated siRNAs or treated with 2-aminobicyclo-(2,2,1)-heptanecarboxylic acid (BCH), a SLC3A2/LAT1 transporter inhibitor, for 96 h. Cell viability was measured by incubating cells with water-soluble tetrazolium salt (WST) (Dail Lab) for 1 h at 37 °C. The plate was shaken for 1 min and then aqueous layer in each well was transferred into 96-well plate. The absorbance was measured at 450/650 nm as reported previously^12^.

### Luciferase reporter assay

Huh7.5 cells were cotransfected with a pRL-HL plasmid containing the *Renilla* luciferase gene under the cytomegalovirus (CMV) promoter and the firefly luciferase gene under the control of the HCV IRES and the plasmid indicated in the figures together with the pCH110 reference plasmid. After 48 h after transfection, cells were harvested and then dual-luciferase assays were performed as we described previously^23^.

### HCV pseudoparticle entry assay

HCV pseudoparticles (HCVpp) with E1 and E2 glycoproteins derived from genotype 1a (H77) or genotype 2a (JFH1) and vesicular stomatitis virus pseudoparticles (VSVpp) were generated as we described previously^23^. Briefly, approximately 2.5 × 10^6^ HEK293T cells were transfected with 2.5 μg of HCV E1E2 or VSV G envelope expressing plasmid, 7.75 μg of Gag-Pol (polymerase) packaging plasmid, and 7.75 μg of transfer vector encoding the firefly luciferase reporter protein by using polyethyleneimine (Sigma-Aldrich). At 48 h after transfection, supernatants containing HCVpp or VSVpp were collected. For the infection assay, Huh7.5 cells were transfected with siRNAs for 48 h and then infected with either HCVpp or VSVpp for 6 h. Cells were then replaced with fresh culture media. At 48 h postinfection, cells were harvested and luciferase activity was determined.

### Binding and entry assays

Approximately 0.6 × 10^5^ Huh7.5 cells seeded on 12-well plate were transfected with either negative control siRNA or SLC3A2 siRNA for 48 h. For HCV binding assay, cells were incubated with Jc1 at 4 °C for 2 h to allow virions for binding but not to internalize the target cells. After washing cells with PBS, bound virions were measured by qRT-PCR. For HCV entry assay, cells were also incubated with Jc1 at 4 °C for 2 h, washed with PBS and then temperature was shifted to 37 °C for 4 h to allow virions to internalize the cells. Cells were then trypsinized and washed twice with PBS to remove non-internalized virions. HCV entry was indirectly determined by analyzing the intracellular HCV RNA levels by qRT-PCR^12,23^.

### Quantification of RNA

Quantitative real-time PCR (qRT-PCR) experiments were performed as we reported previously^12,23^.

### Single-cycle HCV infection

Single-cycle infectious HCV (HCVsc) was generated from a replicon *trans*-packaging system as previously described^23^. Briefly, Huh7.5 cells were cotransfected with pHH/SGR-Luc plasmid which carries a bicistronic HCV subgenomic replicon (NS3-NS5) firefly luciferase reporter with a Pol I promoter/terminator, and a HCV core-NS2 expression plasmid by using Lipofectamine 2000 reagent (Invitrogen, Carlsbad, CA) according to the manufacturer’s protocol. Culture medium containing HCV-like infectious particle (HCV-LP) was collected at 5 days after transfection. For single-cycle HCV infection assay, Huh7.5 cells were pretreated with either negative control siRNA or siRNA targeting SLC3A2 for 48 h and then infected with HCVsc. At 48 h postinfection, cells were harvested and luciferase activity was determined.

### Immunoprecipitation

HEK293T cells were cotransfected with V5-tagged E2 and either Flag-tagged SLC3A2 or Flag-tagged LAT1. Total amounts of DNA were adjusted by adding an empty vector. At 30 h after transfection, cells were harvested and immunoprecipitation assay was performed as we described previously^12,23^. Cells were lysed in buffer containing 140 mM NaCl, 2.7 mM KCl, 10 mM Na_2_HPO_4_, 1.8 mM KH_2_PO_4_, 0.2% NP-40, and protease inhibitor cocktail, and then incubated for 10 min on ice. Cell lysates were centrifuged at 13,000 rpm for 10 min. The supernatant was then incubated overnight at 4 °C with the indicated antibody. The samples were further precipitated with 40 μl of protein A beads (Sigma-Aldrich) for 1 h at 4 °C. The beads were subsequently washed five times in washing buffer, and then bound proteins were detected by immunoblot analysis.

### SLC3A2/LAT1 transporter inhibitor assay

SLC3A2/LAT1 transporter inhibitor, 2-aminobicyclo-(2,2,1)-heptane-carboxylic acid (BCH), was dissolved in 1N NH_4_OH and stored at room temperature. Final concentration of NH_4_OH in the medium was 200 μM. Huh7.5 cells were pre-incubated with various amounts of BCH in 1 h and then infected with Jc1 in the presence of BCH. At 48 h postinfection, cells were harvested and both RNA and protein levels were determined.

### Cell proliferation assay

Cell proliferation was determined by WST assay. Huh7.5 cells were either mock-infected or infected with Jc1 for 4 days and then treated with either NH_4_OH (vehicle) or BCH. Cells were re-plated onto 96-well plates at a density of ~2 × 10^3^ cells per well. At the indicated time intervals after chemical treatment, 10 μL of WST solution was added to each well to determine cell growth.

### Analysis of GDH enzymatic activity

Leucine stimulates GDH activity in hepatocytes^40^. In order to measure L-leucine uptake in Huh7.5 cells, the enzymatic activity of GDH was analyzed by using a GDH Activity Colorimetric Assay kit (BioVision, Catalog #K729-100) according to the manufacturer’s instructions. GDH activity was measured OD at 450 nm in a VersaMax Microplate Reader (Molecular Devices USA).

### Statistical analysis

Data are presented as means ± standard deviations (SDs). Student’s *t* test was used for statistical analysis. The asterisks on the figures indicate significant differences (*P < 0.05; **P < 0.01; ***P < 0.001; ns, not significant).
